# Poor Sleep Quality and Its Consequences on Mental Health During the COVID-19 Lockdown in Italy

**DOI:** 10.3389/fpsyg.2020.574475

**Published:** 2020-11-09

**Authors:** Christian Franceschini, Alessandro Musetti, Corrado Zenesini, Laura Palagini, Serena Scarpelli, Maria Catena Quattropani, Vittorio Lenzo, Maria Francesca Freda, Daniela Lemmo, Elena Vegni, Lidia Borghi, Emanuela Saita, Roberto Cattivelli, Luigi De Gennaro, Giuseppe Plazzi, Dieter Riemann, Gianluca Castelnuovo

**Affiliations:** ^1^Department of Medicine and Surgery, University of Parma, Parma, Italy; ^2^Department of Humanities, Social Sciences and Cultural Industries, University of Parma, Parma, Italy; ^3^IRCCS Istituto delle Scienze Neurologiche di Bologna, Bologna, Italy; ^4^Department of Clinical and Experimental Medicine, Psychiatric Section, University of Pisa, Azienda Ospedaliera Universitaria Pisana (AOUP), Pisa, Italy; ^5^IRCCS Fondazione Santa Lucia, Rome, Italy; ^6^Department of Clinical and Experimental Medicine, University of Messina, Messina, Italy; ^7^Department of Humanistic Studies, University of Naples Federico II, Naples, Italy; ^8^Department of Health Sciences, University of Milan, Milan, Italy; ^9^Department of Psychology, Catholic University of Milan, Milan, Italy; ^10^Psychology Research Laboratory, Ospedale San Giuseppe, Istituto Auxologico Italiano IRCCS, Verbania, Italy; ^11^Department of Biomedical, Metabolic and Neural Sciences, University of Modena and Reggio Emilia, Modena, Italy; ^12^Medical Center, Department of Psychiatry and Psychotherapy, Faculty of Medicine, University of Freiburg, Freiburg, Germany

**Keywords:** sleep quality, sleep habits, COVID-19, Italian lockdown, depression, anxiety, stress, clinical psychology

## Abstract

**Background:**

Coronavirus disease 2019 (COVID-19) seriously affected the whole of Italy. The extreme virulence and the speed of propagation resulted in restrictions and home confinement. This change was immediately perceived by people who found themselves exposed to feelings of uncertainty, fear, anger, stress, and a drastic change in the diurnal but above all nocturnal lifestyle. For these reasons, we aimed to study the quality of sleep and its connection to distress levels and to evaluate how lifestyle changed in the Italian population during the lockdown.

**Methods:**

By means of an Internet survey we recruited 6,519 adults during the whole of the COVID-19 lockdown (from March 10–1st phase to May 4–2nd phase). We investigated the sociodemographic and COVID-19-related information and assessed sleep quality using the Medical Outcomes Study–sleep scale (MOS-SS) and mental health with the short form of Depression, Anxiety, and Stress Scales–21 Items (DASS-21). Multiple logistic regression model was used to evaluate the multivariate association between the dependent variable (good sleeper vs. poor sleeper) and all the variables that were significant in the univariate analysis.

**Results:**

A total of 3,562 (55.32%) participants reported poor sleep quality according to the MOS-Sleep Index II score. The multiple binary logistic regression results of poor sleepers revealed several risk factors during the outbreak restrictions: female gender, living in Central Italy, having someone close who died because of COVID-19, markedly changed sleep–wake rhythms characterized by earlier or postponed habitual bedtime, earlier habitual awakening time and reduced number of afternoon naps, and extremely severe levels of stress, anxiety, and depression.

**Conclusion:**

This is the first study designed to understand sleep quality and sleep habits during the whole of the lockdown period in the Italian population that provides more than 6,000 participants in a survey developed specifically for the health emergency related to COVID-19. Our study found that more than half of the Italian population had impaired sleep quality and sleep habits due to elevated psychological distress during the COVID-19 lockdown containment measures. A multidisciplinary action should be undertaken in order to plan appropriate responses to the current crisis caused by the lockdown for the COVID-19 outbreak.

## Introduction

Coronavirus disease 2019 (COVID-19) was first identified as severe acute respiratory syndrome coronavirus 2 (SARS-CoV-2) in December 2019 by healthcare professionals in Wuhan City (China). Since then, it has spread rapidly throughout Hubei Province and other areas in China soon becoming a worldwide health problem affecting over 100 nations. During the same period, the World Health Organization indicated “COVID-19” as the official name to refer to the severe acute respiratory syndrome caused by SARS-CoV-2 (World Health Organization, 2020). Italy was the most seriously impacted country in Europe.

The Italian prime minister introduced the “I stay home” government decree (Italian Ministry of Health, 2020), which concerned the entire country and formally ordered people to stay at home. Travel restrictions, lockdown of schools and workplaces were the key measures of the “I stay home” decree together with the use of protective devices.

It is beyond doubt that this decision helped to prevent the further spread of the virus and was a necessary imposition to limit the number of patients being admitted to hospital. Nevertheless, from a psychological point of view, people undergoing this first form of intervention were exposed to feelings of uncertainty, fear, anger, and frustration that may easily lead to anxiety, boredom, and/or uneasiness (Brooks et al., 2020; De Giorgio, 2020; Holmes et al., 2020; Rubin and Wessely, 2020; Wang et al., 2020a).

This new situation where people were forced to manage work or study at home, with all the burden of worries stemming from almost inevitable health risks and social distancing, has had a strong impact on daily functioning and night-time sleep (Altena et al., 2020).

Sleep and stress have been described in a bidirectional relationship across the life span (Lo Martire et al., 2019) with stressors impacting on sleep quality and vice versa. In particular, high levels of cognitive and physiological arousal in response to stressors have been hypothesized to interfere with sleep according to the hyperarousal model of insomnia within the framework of a self-reinforcing loop (Morin et al., 1993, 2002; Harvey, 2002; Morin and Espie, 2003; Harvey et al., 2005, 2014; Bonnet and Arand, 2010; Riemann et al., 2010, 2015). In this difficult period, vigilance was constantly high: feelings of loss, excessive use of the Internet to seek information or to maintain social relationships, worries about getting infected, impulsive decisions, and rigid expectations were just some of the factors that could have interfered with a good sleep quality in the Italian population. Moreover, quarantine could have reduced the daylight exposure, which is essential for synchronizing the circadian body clock, consequently affecting many processes including sleep and mood (McClung, 2013; Vadnie and McClung, 2017). Hence, the current study aimed to investigate the quality of sleep and the lifestyle changes in the Italian population during the lockdown imposed by the “I stay home” decree-law issued by the Italian government (March 10) to its end (May 4–the so-called 2nd phase) expecting to find disrupted sleep.

In addition, since sleep quality is well known as being a crucial element of psychological health and a disturbed sleep has been related to psychopathology (Hertenstein et al., 2019), we wanted to study levels of anxiety, depression, and stress in the Italian population in relation to the quality of sleep experienced during the lockdown.

## Materials and Methods

### Study Design

We conducted a cross-sectional study using a short sociodemographic and COVID-19-related information chart and self-administered questionnaire delivered by means of an Internet survey. Data were collected from the issue of the “I stay home” Italian government decree-law (“1st phase” –total lockdown) on March 10 to May 4, 2020 (“2nd phase” –end of lockdown).

### Ethics Statement

This study was conducted in accordance with the Declaration of Helsinki, and the study protocol was approved by the Ethics Committee of the Center for Research and Psychological Intervention (CERIP) of the University of Messina. Electronic informed consent was obtained from each participant prior to starting the investigation. Participants could withdraw from the survey at any moment without needing to provide a reason.

### Procedure and Participants

The data were collected through an online survey (conducted with Microsoft Azure). On the Microsoft Azure platform, information and consent to the processing of personal data were prepared, and furthermore, consent was requested to provide an email contact; the subject was asked to create an identification code in order to anonymize it. Only after expressing consent the URL of the Google Form (Google Form) was available, and the subject needed to affix the identification code previously created to secure anonymity for all the partners and collaborators involved in this multicenter project. Questionnaires were created on the Google Cloud platform, which was anonymous. The survey study was advertised via university communication systems as well as online forums (e.g., through virtual learning environments and Facebook accounts) or WeChat groups. Our questionnaire was set to proceed only when each option was completed before the final submission.

The study surveyed a convenience sample of 6,519 adults from 18 years old or above who lived in Italy and were recruited via notices in several Italian universities (University of Parma; University of Messina; Catholic University of Milan; University of Milan, La Statale; University of Napoli, Federico II). People who do not live in Italy or participants who do not complete all the questionnaires were excluded (*n* = 43 and *n* = 37, respectively).

Data reported in the current study were part of a wider project called “Resilience and the COVID-19: how to react to perceived stress. Effects on sleep quality and diurnal behavior/thoughts.” This multipurpose project was designed to investigate the impact of lockdown in the Italian population.

### Measures

#### Demographic Information

The variables examined in the sociodemographic section included gender, age, marital status (single or not), education level (secondary education or higher), occupation (employed or unemployed), region of origin, and general information about family and home (having or not children, number of people living in the house, size of the house, presence/absence of garden or balcony).

According to the Italian Institute of Statistics (ISTAT), the Italian territory can be divided into three macro-areas: North (1): Valle D’Aosta, Piedmont, Lombardy, Liguria, Emilia Romagna, Friuli-Venezia Giulia, Trentino-Alto Adige, Veneto; Center (2): Lazio, Marche, Tuscany and Umbria; South (3) Abruzzo, Molise, Campania, Apulia, Basilicata, Calabria, Sardinia, and Sicily.

#### Work-Related Data

This section assessed information on participants’ employment data and any changes that occurred following the onset of the lockdown due to the COVID-19 pandemic: occupation, presence/absence of public contacts on workplace, working or not after the onset of COVID-19 pandemic, and information about work changes (at the office or smart working; number of hours: increased/reduced or loss of work).

The classification of occupations is based on an official list (“Nomenclatura e Classificazione delle Unità Professionali–CP2011, ISTAT). Moreover, we added the following categories: unemployed or job-seekers; retired persons; working students or not, and last, we extrapolated the health professions from the ISTAT categories.

#### Issues Related to COVID-19 Data

We collected data about participants and their relatives/friends’ possible COVID-19 contacts/infection and the effects that the new emergency had on their social relationships (decreases/improvements of face-to-face or online contacts) with *ad hoc* items according to the Chinese findings (Wang et al., 2020b; Zhang et al., 2020): positivity or not to the COVID-19 virus, having been forced or not to stay in an obligatory quarantine, having people close who tested positive or not, having lost someone close due to COVID-19, and possible changes in psychical or online relationships (from a decrease to an improvement).

#### Sleep-Related Data

The participants’ sleep habits during the lockdown period were assessed. In particular, we evaluated the changes in the habitual bedtime, awakening time, and napping. Answers regarding bedtime and waking time ranged from bringing forward to postponing the usual time. Instead, for the napping time, the answers were aimed at understanding if they were increased or reduced compared to usual.

#### The Medical Outcomes Study–Sleep Scale (MOS-SS)

The sleep quality of the Italian population during the lockdown period was assessed using the Medical Outcomes Study–Sleep Scale (MOS-SS) (Hays and Stewart, 1992). The MOS-SS is a self-administered validated instrument with 12 self-reported questions to determine sleep quality and quantity within a 4-week period. We decided to adopt only the global index of MOS-SS to assess the quality of sleep defined as Sleep Index II (score range from 0 to 100), with higher scores indicating greater sleep problems. A cut-off scoring of 25.8 (Hays et al., 2005; Rejas et al., 2007; Martin et al., 2009) is considered as having poor sleep. The Italian version is available (Palagini and Manni, 2016). In this study, reliability of Sleep Problem II index scales was good, with a Cronbach’s α of 0.85.

#### The Depression Anxiety Stress Scale–21 (DASS-21)

Symptoms of common mental health status were assessed using the short form of Depression, Anxiety, and Stress Scales–21 Items (DASS-21) (Lovibond and Lovibond, 1995). The DASS-21 is a self-report measure in which participants rate the frequency and severity of depression, anxiety, and stress (emotional reactions).

As measured by the DASS-21, depression assesses dysphoria, anhedonia, lack of incentive, and low self-esteem; anxiety refers to somatic and subjective symptoms of anxiety and an acute response of fear; and stress evaluates irritability, impatience, tension, and persistent arousal.

Subscale scores are calculated as the sum of the responses to the seven items from each subscale multiplied by 2 to suit the original 42 items. The cutoffs for severe depression, anxiety, and stress are ≥21, ≥15, and ≥26, respectively (Lovibond and Lovibond, 1995).

In the current study, the Italian version of DASS-21 showing excellent psychometric properties was adopted (Bottesi et al., 2015).

Excellent levels of reliability were detected in this sample (depression, α = 0.89; anxiety, α = 0.83; stress, α = 0.90).

### Statistical Analysis

Continuous variables were presented as mean and standard deviation (SD), while categorical variables as absolute (n) and relative frequency (%). Chi-square test was used to evaluate the univariate association between MOS-Sleep Index II (sleep disturbance vs. no sleep disturbance) and all the variables described in the section “Materials and Methods.” Multiple logistic regression model was used to evaluate the multivariate association between the dependent variable (good sleeper vs. poor sleeper) and all the variables that were significant in the univariate analysis. The results were presented as odds ratio (OR) with 95% confidence interval (95% CI). The false discovery rate (FDR) correction was applied to adjust the statistical significance to account for multiple comparison (adjusted critic *p* = 0.008) (Benjamini and Hochberg, 1995).

Cronbach’s alpha was used to evaluate the reliability of the questionnaires used in the survey.

Statistical analysis was performed using the statistical package Stata SE, 14.2.

## Results

Our study sample consisted of 6,439 participants: 4,707 (73.1%) females and 1,732 (26.9%) males. The mean age of the sample was 33.9 (SD = 27.6; range 18–82 years), and most of the participants were living in Northern Italy (67.4%). Less than half of the samples had a high school diploma (46.9%) and 28.7% were students. With regard to marital status, 34.9% were unmarried and 28.6% had children.

**FIGURE 1:**
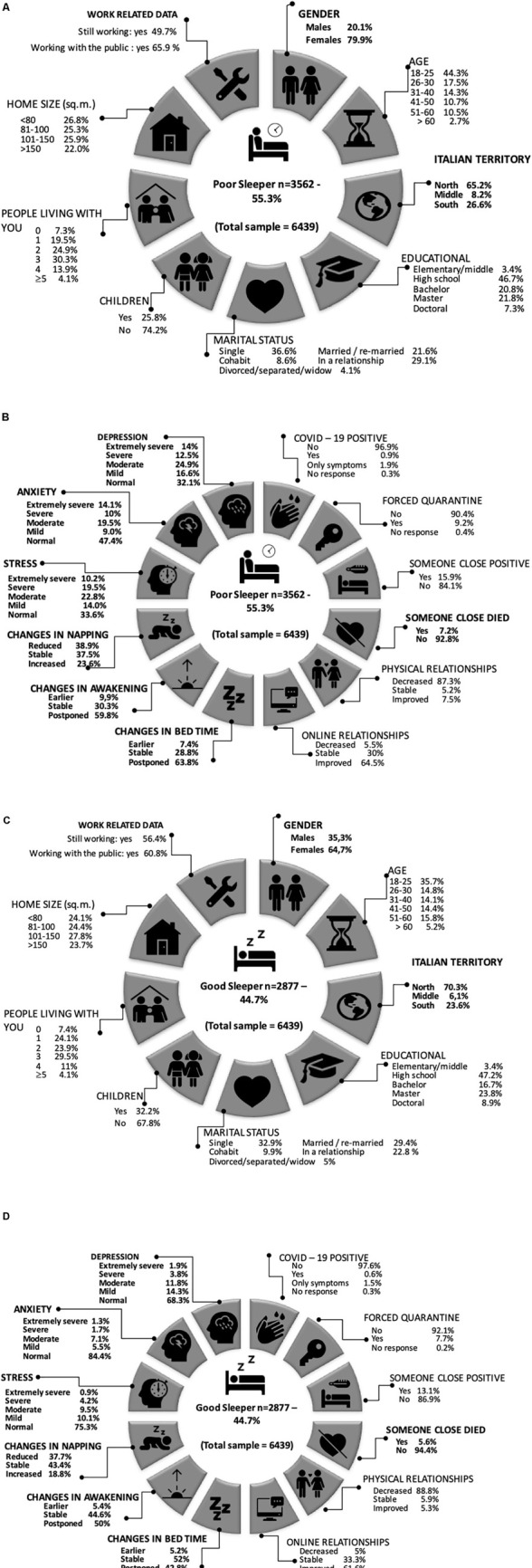
**(A)** Percentages of poor sleeper stratified for the variables. In bold the significations in the multivariable logistic regression model. The icons are selected from the https://icons8.it. **(B)** Percentages of poor sleeper stratified for the variables. In bold the significations in the multivariable logistic regression model. The icons are selected from the https://icons8.it. **(C)** Percentages of good sleeper stratified for the variables. In bold the significations in the multivariable logistic regression model. The icons are selected from the https://icons8.it. **(D)** Percentages of good sleeper stratified for the variables. In bold the significations in the multivariable logistic regression model. The icons are selected from the https://icons8.it.

In Tables 1A,B, we show all the characteristics and differences of the good sleeper and poor sleeper groups in terms of demographic data, living situation during the COVID-19 outbreak, COVID-19 outbreak-related questions, sleep-related data, and mental health in terms of stress, anxiety, and depression. There were 6,439 participants in our study. A total of 3,562 (55.32%) participants reported poor sleep quality according to the MOS-Sleep Index II score. All participants were divided into two groups, poor sleeper (MOS-Sleep Index II total score ≥25.8) and good sleeper (MOS-Sleep Index II total score <25.8). In Figures 1A,B, we show the graphical representation of percentages of poor sleepers stratified for the variables. Also, in Figures 1C,D we show the graphical representation of percentages of good sleepers stratified for the variables.

**TABLE 1-A:** Comparisons between the good sleeper vs. poor sleeper sample with chi-square tests on demographic data and living situations.

	Good sleeper (*n* = 2,877)	Poor sleeper (*n* = 3,562)	*P*
**Demographic data**			
***Gender, n (%)***			< 0.001
Males	1,017(58.7)	715 (41.3)	
Females	1,860(39.5)	2,847(60.5)	
***Age (years old), n (%)***			
18–25	1,026(39.4)	1,579(60.6)	< 0.001
26–30	427 (40.7)	622 (59.3)	
31–40	406 (44.3)	511 (55.7)	
41–50	413 (52.1)	380 (47.9)	
51–60	455 (54.9)	374 (45.1)	
>60	150 (61.0)	96 (39.0)	
***Italian territory, n (%)***			
North	2,022(46.6)	2,321(53.4)	< 0.001
Center	175 (37.5)	292 (62.5)	
South	680 (41.7)	949 (58.3)	
***Education, n (%)***			
Elementary/middle school	99 (45.0)	121 (55.0)	< 0.001
High school	1,358(44.9)	1,663(55.1)	
Bachelor’s degree	480 (39.3)	741 (60.7)	
Master’s degree	685 (46.9)	775 (53.1)	
Doctoral degree	255 (49.3)	262 (50.7)	
***Marital status n (%)***			
Single	946 (42.1)	1,303(57.9)	< 0.001
Married or re-married	845 (52.3)	771 (47.7)	
Cohabitant	285 (48.1)	305 (51.9)	
In a relationship	657 (38.8)	1,035(61.2)	
Divorced/separated/widowed	144 (49.3)	148 (50.7)	
***Children (yes), n (%)***			< 0.001
Yes	925 (50.2)	919 (49.8)	
No	1,952(42.5)	2,643(57.5)	
***People living with you, n (%)***			
0	213 (45.0)	260 (55.0)	< 0.001
1	694 (49.9)	695 (50.1)	
2	687 (43.6)	887 (56.4)	
3	848 (44.0)	1,079(56.0)	
4	318 (39.0)	496 (61.1)	
5+	117 (44.7)	145 (55.3)	
***Home size (sq. m.), n (%)***			
≤80	693 (42.1)	954 (57.9)	< 0.001
81–100	703 (43.8)	902 (56.2)	
101–150	800 (46.5)	922 (53.5)	
>150	681 (46.5)	784 (53.5)	
***Having a Garden or Balcony, n (%)***			
Yes	2,624(45.1)	3,198(54.9)	< 0.001
No	253 (41.0)	364 (59.0)	
**Work-related data**			
***Working with the public, n (%)***			< 0.001
Yes	1,749(42.7)	2,347(57.3)	
No	1,128(48.1)	1,215(51.9)	
***Still working (yes), n (%)***			< 0.001
Yes	1,622(47.8)	1,772(52.2)	
No	1,255(41.2)	1,790(58,8)	
***Work modality, n (%)***			
Only in office	343 (47.6)	378 (52.4)	0.033
Only through smart working	991 (45.5)	1,187(54.5)	
In office and through smart working	138 (54.8)	114 (45.2)	
Mostly in office	58 (50.4)	57 (49.6)	
Mostly through smart working	142 (51.1)	136 (48.9)	
***Consequences on work, n (%)***			
Increased working hours	340 (45.8)	403 (54.2)	0.582
Work interruption	687 (44.8)	846 (55.2)	
Stable, with same starting time	60 (45.5)	72 (54.5)	
Stable, with earlier starting time	532 (45.7)	633 (54.3)	
Stable, with postponed starting time	189 (48.6)	200 (51.4)	
Reduced working hours	577 (43.5)	751 (56.5)	
Unemployed	502 (42.9)	669 (57.1)	
Other	1 (50.0)	1 (50.0)	
***Occupation, n (%)***			
Retired	69 (59.9)	48 (41.0)	< 0.001
Student	717 (38.8)	1,133(61.2)	
Working student	317 (37.2)	534 (62.8)	
Healthcare employee (public/private)	150 (40.4)	221 (59.6)	
Police/military	33 (62.3)	20 (37.7)	
Artisan, laborer, farmer	59 (58.4)	42 (41.6)	
Employee/manager/owner of business activity	298 (49.3)	306 (50.7)	
Employee/manager/owner of industrial activity	241 (59.2)	166 (40.8)	
Intellectual profession	255 (47.7)	280 (52.3)	
Unemployed/searching	108 (36.2)	190 (63.8)	
Office executive job	21 (60.0)	14 (40.0)	
Technical profession	175 (54.0)	149 (46.0)	
Non-qualified profession	381 (48.3)	408 (51.7)	
Other	53 (51.0)	51 (49.0)	

**TABLE 1-B:** Comparisons between the Good Sleeper vs. Poor Sleeper sample with chi-square tests on COVID-19 related data, sleep habits and mental healths.

	Good sleeper (*n* = 2,877)	Poor Sleeper (*n* = 3,562)	*P*
**COVID-19-related data**			
***COVID-19 positive, n (%)***			
No	2,803(44.9)	3,431(55.1)	0.303
Yes	18 (34.6)	33 (65.4)	
Had symptoms but no swab test	42 (38.9)	66 (61.1)	
No answer/other	8 (30.4)	10 (69.6)	
***Forced quarantine, n (%)***			
No	2,649(45.1)	3,219(54.9)	0.037
Yes	221 (40.2)	329 (59.8)	
No answer	7 (33.3)	14 (66.7)	
***Someone close positive, n (%)***			0.002
Yes	377 (40.0)	566 (60.0)	
No	2,500(45.5)	2,996(54,5)	
***Someone close died, n (%)***			
Yes	160 (38.3)	258 (61.7)	0.004
No	2,717(45.1)	3,304(54.9)	
***Changes in physical relationships, n (%)***			
Decreased	2.554 (45.1)	3,108(54.9)	< 0.001
Stable	171 (47.8)	187 (52.2)	
Improved	152 (36.3)	267 (63.7)	
***Changes in online relationships, n (%)***			
Decreased	147 (42.7)	197 (52.3)	0.020
Stable	958 (47.3)	1,068(52.7)	
Improved	1,772(43.5)	2,297(56.5)	
**Sleep-related data**			
***Changes in the habitual bedtime, n (%)***			
Earlier	151 (36.3)	265 (63.7)	< 0.001
Stable	1,494(59.3)	1,025(40.7)	
Postponed	1,232(35.2)	2,272(64.8)	
***Changes in the habitual awakening time*, *n (%)***			
Earlier	154 (30.3)	354 (69.7)	< 0.001
Stable	1,284(54.3)	1,079(45.7)	
Postponed	1,439(40.3)	2,129(59.7)	
***Changes in the habitual napping, n (%)***			
Reduced	1,085(43.9)	1,387(56.1)	< 0.001
Stable	1,249(48.3)	1,336(51.7)	
Increased	542 (39.2)	839 (60.8)	
**Mental Health**			
***Stress, n (%)***			
Extremely severe	25 (6.4)	362 (93.6)	< 0.001
Severe	121 (14.9)	694 (85.1)	
Moderate	274 (25.2)	811 (74.8)	
Mild	291 (36.9)	498 (63.1)	
Normal	2,166(64.4)	1,197(35.6)	
***Anxiety, n (%)***			
Extremely severe	38 (7.9)	504 (92.1)	< 0.001
Severe	49 (12.1)	355 (87.9)	
Moderate	203 (22.7)	693 (77.3)	
Mild	159 (33.2)	320 (66.8)	
Normal	2,428(59.0)	1,690(41.0)	
***Depression, n (%)***			
Extremely severe	55 (10.0)	497 (90.0)	< 0.001
Severe	107 (19.4)	444 (80.6)	
Moderate	338 (27.6)	888 (72.4)	
Mild	411 (41.1)	590 (58.9)	
Normal	1,966(63.2)	1,143(36.8)	

***(A,B)** show frequencies and percentages of good and poor sleepers for all investigated variables.*

Sixty-seven participants who gave no answers to questions about forced quarantine or being positive to COVID-19 were removed to perform the multivariate logistic regression. Table 2 shows the multiple binary logistic regression results of poor sleepers. Significant factors were found in female gender (OR: 1.66; 95% CI: 1.45–1.90), living in Central Italy (OR: 1.39; 95% CI: 1.10–1.76), having someone close who died due to COVID-19 (OR: 1.41; 95% CI: 1.09–1.81), earlier (OR: 1.59; 95% CI: 1.22–2.07) or postponed (OR: 2.10; 95% CI: 1.81–2.41) habitual bedtime, earlier habitual awakening time (OR: 1.47; 95% CI: 1.14–1.90), reduced number of afternoon naps (OR: 1.32; 95% CI: 1.13–1.56), experiencing mild (OR: 2.10; 95% CI: 1.76–2.52), moderate (OR: 2.60;95% CI: 2.16–3.16), severe (OR: 3.37; 95% CI: 2.59–4.39), or extremely severe (OR: 4.83; 95% CI: 2.95–7.92) stress, having mild (OR: 1.61; 95% CI: 1.29–2.02), moderate (OR: 2.39; 95% CI: 1.98–2.89), severe (OR: 3.11; 95% CI: 2.23–4.53), or extremely severe (OR: 3.74; 95% CI: 2.54–5.51) anxiety, and having mild (OR: 1.53; 95% CI: 1.30–1.81), moderate (OR: 1.67; 95% CI: 1.38–1.98), severe (OR: 1.77; 95% CI: 1.32–2.30), or extremely severe (OR: 2.39; 95% CI: 1.69–3.53) depression. Furthermore, being positive to COVID-19 was almost significant (OR: 1.96; *p* = 0.066).

**TABLE 2:** Multivariable logistic regression analysis of the poor sleeper sample.

Variable	OR	*P*	95% CI
**Demographic data**			
Gender (female)	1.66	<0.001	1.45–1.90
**Age**			
18–25	1.04	0.764	0.67–1.70
26–30	1.20	0.920	0.79–1.91
31–40	1.17	0.458	0.79–1.77
41–50	0.98	0.920	0.66–1.49
51–60	1.06	0.764	0.72–1.55
>60	Reference		
**Italian territory**			
North	Reference		
Center	1.39	0.006	1.10–1.76
South	1.13	0.104	0.95–1.31
**Education level**			
Elementary/middle school	Reference		
High school	0.86	0.394	0.61–1.21
Bachelor’s degree	0.95	0.826	0.67–1.38
Master’s degree	0.95	0.765	0.66–1.36
Doctoral degree	0.89	0.594	0.59–1.35
**Marital status**			
Single	Reference		
Married or re-married	0.93	0.603	0.72–1.21
Cohabit	0.84	0.183	0.65–1.09
In a relationship	0.89	0.155	0.77–1.04
Divorced/separated/widowed	1.00	0.991	0.70–1.43
Children (no)	1.31	0.037	1.01–1.69
**People living with you**			
0	Reference		
1	0.80	0.119	0.61–1.06
2	0.91	0.527	0.69–1.21
3	0.88	0.372	0.66–1.17
4	1.10	0.553	0.80–1.51
5+	0.81	0.293	0.54–1.20
**Home size (sq. m.)**			
≤80	Reference		
81–100	1.00	0.995	0.84–1.19
101–150	0.88	0.170	0.74–1.05
>150	0.89	0.223	0.73–1.07
Having a garden or balcony (yes)	0.89	0.266	0.72–1.09
Working with the public (yes)	1.12	0.085	0.98–1.28
Still working (yes)	0.99	0.883	0.86–1.13
**Occupation**			
Retired	Reference		
Student	1.04	0.897	0.59–1.81
Working student	1.00	0.973	0.57–1.78
Healthcare employee (public/private)	1.37	0.287	0.77–2.43
Police/military	0.93	0.856	0.40–2.12
Artisan, laborer, farmer	0.65	0.226	0.32–1.31
Employee/manager/owner of business activity	0.88	0.637	0.50–1.52
Employee/manager/owner of industrial activity	0.81	0.479	0.46–1.44
Intellectual profession	0.83	0.506	0.47–1.45
Unemployed/searching	1.29	0.398	0.72–2.32
Office executive job	0.83	0.687	0.32–2.10
Technical profession	0.77	0.367	0.43–1.37
Non-qualified profession	0.89	0.687	0.52–1.54
Other	0.73	0.372	0.37–1.45
**COVID-19 positive**			
No	Reference		
Yes	1.96	0.066	0.95–4.03
Had symptoms but no swab test	1.36	0.196	0.85–2.20
**Forced quarantine**			
No	Reference		
Yes	0.88	0.270	0.70–1.10
Someone close positive (yes)	0.97	0.749	0.81–1.16
Someone close died (yes)	1.41	0.008	1.09–1.81
**Changes in physical relationships**			
Decreased	Reference		
Stable	1.10	0.478	0.85–1.42
Improved	1.18	0.178	0.93–1.52
**Changes in online relationships**			
Decreased	Reference		
Stable	0.97	0.838	0.73–1.29
Improved	1.02	0.900	0.78–1.33
**Changes in the habitual bedtime**			
Earlier	1.59	0.001	1.22–2.07
Stable	Reference		
Postponed	2.10	<0.001	1.81–2.41
**Changes in the habitual awakening time**			
Earlier	1.47	0.004	1.14–1.90
Stable	Reference		
Postponed	1.01	0.735	0.88–1.17
**Changes in the habitual napping**			
Reduced	1.32	0.001	1.13–1.56
Stable	Reference		
Increased	1.11	0.114	0.94–1.28
**Stress**			
Extremely severe	4.83	<0.001	2.95–7.92
Severe	3.37	<0.001	2.59–4.39
Moderate	2.60	<0.001	2.16–3.16
Mild	2.10	<0.001	1.76–2.52
Normal	Reference		
**Anxiety**			
Extremely severe	3.74	<0.001	2.54–5.51
Severe	3.11	<0.001	2.23–4.35
Moderate	2.39	<0.001	1.98–2.89
Mild	1.61	<0.001	1.29–2.02
Normal	Reference		
**Depression**			
Extremely severe	2.39	<0.001	1.69–3.53
Severe	1.77	<0.001	1.32–2.30
Moderate	1.67	<0.001	1.38–1.98
Mild	1.53	<0.001	1.30–1.81
Normal	Reference		

## Discussion

As far as we know, this is the first study to assess sleep quality and its negative consequences on mental health in the Italian population during the whole of the COVID-19 lockdown (from March 10–1st phase to May 42nd phase). This survey interestingly highlights how quarantine and restriction measures worsened sleep habits, leading to a whole series of consequences on people’s health. In particular, our study found that 55.32% of the sample of 6,439 Italian participants experienced disrupted sleep patterns during the outbreak restrictions.

In our study, the poor sleeper group presented with more negative effects on psychological well-being related to the COVID-19 lockdown. We outlined some risk factors for the development of sleep disturbance: female gender (79.9%), living in Central Italy, losing a loved one due to COVID-19, having markedly changed sleep–wake rhythms (specifically, going to bed earlier or later than the usual time), getting up earlier than usual and having increased the habitual napping time, having moderate to very severe stress (22.8–10.2%), anxiety (19.5–14.1%), and depression levels (25.9–14%).

The prevalence rate of poor sleepers in our population is consistent with 52.4 and 57.1% of two Italian resident surveys [lockdown period: from March 17 to 23 (Cellini et al., 2020) and from March 18 to April 2 (Casagrande et al., 2020)] and higher than in China’s general population (36.38%) (online survey from to February 18 to 25) (Zhao et al., 2020) during the outbreak. In addition, our sample with sleep problems reported a remarkable alteration in their sleep habits: 63.8% reported postponing or bringing forward (7.4%) bedtime; 59.8% reported the need for delayed awakening and an increased napping time in 23.6% during the daytime. All these lifestyle changes seem to be followed by worrying symptoms, such as altered sleep–wake rhythms, which can be interpreted by the negative psychosocial changes observed by Brooks et al. (2020), especially in sleep habits during the COVID-19 outbreak. Evidence (Lo Martire et al., 2019) shows that temporally close excessive and unpredictable stress can impact on the defense system and the central nervous system: stress is modulated by the individual’s psychological responses, which include neuro-endocrine and behavioral components, such as changes in the activity and immune function of the hypothalamic pituitary adrenal (HPA) axis. Consequently, the activation of the HPA system by stress is incompatible with physiological sleep leading to lower sleep quality, longer sleep latency, increased awareness during the night, and more sleep complaints (Tempesta et al., 2013). On the other hand, impairment of sleep quality is a common behavioral consequence of the acute and chronic response to sleep, stress, and trauma (Lo Martire et al., 2019) and determines further increases in the HPA system, thereby promoting a vicious cycle of stress and worsened sleep quality (Lo Martire et al., 2019).

In addition, our results showed that sleep habits affected by quarantine had a strong impact on sleep quality: individuals who go to bed earlier or after the usual time and wake up earlier than usual or have increased habitual napping time have poor sleep quality. Regular schedules also played a role: there is an association between irregular schedules and the complaint of poor sleep (Pilz et al., 2018). Quality sleep requires regular schedules: numerous studies on the deleterious effects of shift work and social jet-lag prove the negative effects of these disturbed rhythms, both metabolically and psychiatrically (Rutters et al., 2014; Levandovski et al., 2011; Sűdy et al., 2019). The biological clock depends on a strong light signal in the morning to update the central clock (Roenneberg et al., 2013). Exposure to intense light in the evening directly stimulates the arousal systems; thus, the individual remains more vigilant and goes to bed later (Phipps-Nelson et al., 2003). In addition, exposure to light in the evening may affect melatonin secretion, resulting in altered night sleep duration (Gooley et al., 2011). This lag may become confusing for the subject once the confinement measures had been lifted: resetting the clock can be difficult, with severe drowsiness and sleeplessness in the evening.

The present study also found a strong association, also recently documented in the Italian population (Casagrande et al., 2020; Cellini et al., 2020), between those who have poor sleep quality and psychological distress. In particular, our study reveals that those who have high levels of stress, anxiety and depression had a higher probability of have sleep problems.

Since quarantine is characterized by self-isolation, social estrangement, separation, loss of freedom, and uncertainty, negative emotions such as fear, anger, and frustration are common and may lead to anxiety, boredom, and/or a feeling of uneasiness (Brooks et al., 2020; Holmes et al., 2020; Qiu et al., 2020; Roychowdhury, 2020). Such feelings endorse negative beliefs about the individual ability to cope. In this context, Brooks et al. (2020) present the main factors that seem to negatively influence our system during the quarantine period: (1) duration of the quarantine; (2) fear of getting infected/spreading the infection; (3) feelings of frustration and boredom; (4) inadequate supply capacity; and (5) lack of sufficient/salient information (Brooks et al., 2020). Moreover, the impossibility to take part in usual day-to-day activities, like outdoor physical activity or physical contact with others, together with dramatic changes in working modalities might encourage a dysregulation of the wake/sleep cycle (Panahi and Tremblay, 2018) as well as increasing psychological distress (Chirico et al., 2020).

According to the cognitive–behavioral model of insomnia, the 3P model, stress is the most common precipitating factor (Spielman et al., 1987). Therefore, all the highlighted COVID stressors seem to trigger elevated cognitive and physiological hyperarousal in a vicious circle that may have impaired sleep quality. Furthermore, when the perceived stress exceeds an individual’s resources, the consequent change in the emotional state (e.g., anxiety) affects wellbeing (Lazarus and Folkman, 1984; Lazarus et al., 1985). Moreover, recent studies underlined how sleep deprivation (Spiegelhalder et al., 2013) significantly reduces the functional connectivity in frontal brain regions, including the ventromedial regions involved in strategies of decision making based on reward and punishment. These alterations have been related with a loss in emotional control and a general tendency to take impulsive and risky decisions that may contribute to maintaining the mood disorder (Kalmbach et al., 2018).

Furthermore, in line with scientific evidence on sleep disturbances (Ohayon and Smirne, 2002; Terzano et al., 2004; Léger et al., 2008) the female gender is more exposed than the male to having problems with sleep. According to other studies on epidemic and quarantine conditions, in nation-wide pandemic catastrophes, sleep disorders are more present in women than in men (Kendler et al., 2001; McLean and Anderson, 2009). Some evidence (Driver et al., 1996; Baker and Driver, 2004; Dzaja et al., 2005; Zhang and Wing, 2006; Davidson, 2009) shows that the changing hormone profile across the reproductive life of a woman, from puberty through the reproductive period to the postmenopausal years, may have a significant influence on sleep leading to sleep alteration or disruption and other vulnerabilities specific to psychological disorders in women (Soni et al., 2013).

Moreover, in our study, the death of someone close due to COVID-19 seems to be a risk factor in the onset of a sleep disorder, as described in the literature (Healey et al., 1981; Morin et al., 2003; Monk et al., 2008). The death of family members or close friends can be very traumatic, especially when the circumstances are unexpected as in this period. Surely, in this dramatic period, not being able to bid farewell and give loved ones a dignified burial have certainly worsened the grief over the loss, with consequences on sleep.

Paradoxically, in our sample, neither age nor the type of occupation seemed to represent a risk factor in developing sleep disorders, as it has been instead described in the Chinese healthcare staff and attributed to the grueling work shifts and the constant witnessing of death and suffering. Most likely, our sample of healthcare professionals was too small (5.7%) compared not only to the other professions but also to the Chinese sample, since our survey was voluntary and did not involve direct administration (Pappa et al., 2020; Xiao et al., 2020; Xue et al., 2020; Zhang et al., 2020) in the front line (hospitals), where these workers were active. In addition, with age, nighttime sleep becomes more fragmented, and total sleep time is reduced (Ohayon et al., 2017). In this stressful condition where COVID induced profound changes in sleep habits, we may all find ourselves, regardless of age differences, experiencing sleep in a problematic way.

Another interesting result that we reported is a risk of sleep problems in Central Italy, although those who live in Northern Italy have been considered the main Italian core of the emergency, due to the greater number of infections and deaths (Istituto Superiore di Sanità, 2020). Perhaps the worries about personal safety, transmitting the disease to family members, stigmatization from being infected, shift work, and interpersonal isolation can manifest by hyperarousal states, as well as problems with anxiety and stimulus control. Stigma can lead to continued fear as people with a disease anticipate discrimination (Audet et al., 2013), and we know at the same time that stigma can be one of the most powerful barriers to delivering prevention, treatment, and care to the most vulnerable, who are at the same time the ones most in need. Moreover, it is possible to hypothesize that another explanation of this result is due to the fact that residents of this area, following the violent L’Aquila earthquake, have developed greater psychological consequences and are more prone to developing posttraumatic stress disorder symptomatology affecting sleep (Tempesta et al., 2013; Ferrara et al., 2016). Finally, the people living in this location perceive themselves to be at higher risk of infection. In fact, some studies (Alexander and Klein, 2001; Cacciaglia et al., 2017) suggested that people who were repeatedly exposed to traumatic events were prone to suffering from many psychological problems and consequently have sleep disorders.

We believe that multidisciplinary action should be taken in order to plan appropriate responses to the current crisis caused by the COVID-19 health emergency. According to Holmes et al. (2020), the consequences of COVID-19 epidemic in the global population are truly unknown and worrying. Therefore, a range wealth of collaborative work is necessary where psychologists, psychiatrists, neurologists, pulmonologists, and virologists cooperate to finalize a policy that will help the population not only to reduce fear and stigma but also to treat mental health and poor quality of sleep caused by the COVID-19 outbreak (Castelnuovo et al., 2020). In particular, as our results highlight, the poor quality of sleep, especially in predisposed subjects, might represent a risk factor for the development of chronic insomnia or other sleep disorders. Moreover, we confirm the negative psychosocial changes observed by Brooks et al. (2020), especially in sleep habits. These are probably caused not only by factors such as poor exposure to sunlight, reduced physical activity, and psychological distress (Altena et al., 2020) but also by the lack of regular and scheduled activities. Moreover, the European Academy for Cognitive–Behavioral Treatment of Insomnia has developed some useful recommendations for the family or single people adapted from the key points of cognitive–behavioral therapy for insomnia to manage the risks attendant to home confinement and to give practical advice about how to handle sleep problems (Altena et al., 2020).

In Italy, the Italian Associations of Sleep Medicine, a multidisciplinary association of specialists on sleep disorders and their treatments, have started an intensive awareness campaign about sleep problems during the lockdown and developed an online help desk^1^ where the best sleep medicine experts (neurologists, pulmonologists, psychiatrists, and psychologists) respond online to requests of whoever feels the need to improve sleep in this period of time.

As a whole, the results of our study seem to be relevant to outline risk factors for sleep quality in the Italian population during the COVID-19 emergency, but some limitations need to be considered. First, our study is not representative of the sample compared to the Italian population: it was a convenience sample. In addition, the data and results were derived from a cross-sectional design: it was difficult to make cause-and-effect hypotheses. Second, having adopted an online survey limits the generalizability of the results, although it currently represents the only solution for data collection in the time of outbreak. Subsequently, recruitment bias emerged in our sample, which is characterized by a high number of students and women. This aspect should be considered in the interpretation of the results. Third, in this survey we only adopted a self-report questionnaire. Despite the importance of measuring the subjective perception of sleep and distress, semi-objective or objective measures of sleep (such as a sleep diary or actigraphy) and distress would be useful to support our findings.

## Conclusion

This is the first study designed to understand sleep quality and lifestyle in the Italian population during the lockdown period that provides more than 6,000 participants in a survey developed specifically for the health emergency related to COVID-19. Our study found that more than half of the Italian population had impaired sleep quality and sleep habits during the COVID-19 lockdown containment measures.

The related factors included female gender, Italian territory, loss of a loved one due to COVID-19 during the lockdown, changes in sleep habits, and elevated psychological distress. A multidisciplinary intervention for sleep disorders and related psychological discomfort is fundamental with a view to taking action to deal with the current crisis caused by the restrictions adopted to reduce the COVID-19 outbreak and to cope with the eventuality of new lockdown periods.

## Data Availability Statement

The raw data supporting the conclusions of this article will be made available by the authors, without undue reservation.

## Ethics Statement

This study was conducted in accordance with the Declaration of Helsinki and the study protocol was approved by the Ethics Committee of the Center for Research and Psychological Intervention (CERIP) of the University of Messina. Electronic informed consent was obtained from each participant prior to starting the investigation. Participants could withdraw from the survey at any moment without needing to give a reason.

## Author Contributions

CF provided substantial contributions to the conception of the work, deep analysis of the literature, study design, development, and final approval of the manuscript. AM contributed in the design of the study, participated in the development and revision of the work, and agreement for final approval of the manuscript. CZ and SS contributed to data analysis and agreement for final approval of the manuscript. MQ, VL, MF, DL, EV, LB, ES, and RC contributed to the revision of the work and agreement for final approval of the manuscript. LP, LD, DR, GP, and GC contributed to deep revision of the work, with literature analysis and agreement for final approval of the manuscript. All authors contributed to the article and approved the submitted version.

## Conflict of Interest

GP is a consultant and participated in the advisory board for UCB Pharma, Jazz Pharmaceuticals, Bioprojet, Takeda, and Idorsia outside the submitted work. The remaining authors declare that the research was conducted in the absence of any commercial or financial relationships that could be construed as a potential conflict of interest. The handling editor is currently organizing a Research Topic with one of the authors GC.

## Footnotes

1 http://www.sonnomed.it/
